# Abstract of the Proceedings of the Buffalo Medical Association

**Published:** 1858-07

**Authors:** Austin Flint

**Affiliations:** Secretary


### ART. VIII.—*Abstract of Proceedings of the Buffalo Medical Association.*

TUESDAY EVENING, June 1, 1858.

The Association met.

Present—The President, Dr. Wyckoff, in the chair; Drs. Hamilton, Root, Flint, Miner, Rochester, Newman, Strong, Lemon, Wilcox, and Flint, Jr.

The minutes of the last meeting mere read and approved.

*Prof. Flint*, the late president, then read his address, which had been deferred till the present occasion.*

*Dr. Newman* moved that a vote of thanks be rendered to the retiring president for his address, and also for his establishing the precedent in complying with the clause of the by-laws, requiring the retiring president to deliver an address on some medical subject. Seconded and carried.

*Prof. Rochester* wished to report a case of Traumatic Peritonitis, which had lately occurred in his practice. The patient was a little boy, four years of age, who was brought to his office the Wednesday before. He was taken sick on the preceding evening. He then had copious vomiting and purging, with a high fever. On the next day there were evidences of peritonitis, and examination disclosed an extensive bruise on the lower part of the abdomen. On inquiry, it was ascertained that he had been kicked, the Sunday before, by a larger boy, and, as has been stated, was taken ill on the following Tuesday evening. There were some peculiarities in the case, which seemed to throw some doubt on the diagnosis. He had active delirium, the head being thrown back, which induced a suspicion of some affection of the brain, and repeated epistaxis. Prof. Flint visited the patient in consultation and it was decided to be a case of peritonitis. The delirium continued till death, which occurred on the following Monday.

On *post-mortem* examination, the bruise on the abdomen was found to extend through the skin and fasciæ, and a little way into the substance of the external oblique. Evidences of active inflammation were found under the bruise, and general inflammation of the peritoneum, with formations of pus and lymph. The peritoneal covering of the stomach was inflamed, which accounted for the persisting vomiting. Prof. Rochester had never before met with a case of peritonitis resulting from external injury. There was no rupture of any of the abdominal viscera. He inquired if Prof. Hamilton had ever seen a similar case.

---

* This address will be published in a future number of the Journal.

*Prof. Hamilton* replied that he had not.

*Prof. Hamilton* presented a specimen of disease of the breast, which he had lately removed from an unmarried female, æt. 27, and which for a time had excited a suspicion that it was of a cancerous character, but which he now believed not to be. It had been progressing about six months, having first presented itself as a hard tumor, apparently in the substance of the breast; it grew rapidly and the skin soon becoming involved, it sloughed, making a large and irregular opening, from which a mass of fungous flesh protruded. The discharge was ill-conditioned, bloody and highly offensive. Free incisions were now made on the borders of the opening, and poultices were applied in the hope of that the tissue underneath would suppurate and the unhealthy fungus be extruded; but no benefit was derived from this treatment. Gradually the whole breast became involved; the whole feeling hard and nodulated. The imperfect suppuration continued, and the health was rapidly failing; night sweats and hectic having already occurred. Two or three glands in the axilla also became involved. It was now determined to extirpate the breast, and the operation was made by Dr. Hamilton.

The tumor was found to have contracted adhesions to the muscles underneath; and one of the glands in the axilla was followed, and removed also. An examination of the product showed that it was composed chiefly of the glandular structure of the breast, filled with a dense fibro-plastic material. Dr. John Boardman, by whom the tumor was subjected to a careful microscopic examination, could discover nothing like cancer cells. The gland removed from the axilla seemed to be a simple scrofulous enlargement.

The wound was healed kindly, and the health of the patient has been rapidly restored.

Dr. Hamilton had never seen true scirrhus in the breast in a person so young as twenty-seven years; and he was glad to find in the tumor itself, since its removal, such conclusive evidence that this was not cancerous. His experience had led him to think that hard cancer of the breast was a disease of age; and he would suggest that true, hard cancer, indicated the degeneration of age — that it was a cessation of the process of repair, and consequent degeneration of the tissue; and that as the arcus senilis, and other similar fatty degenerations, indicated age, so did hard cancer, and that it was not therefore specific any more than were these latter degenerations.

*Dr. Strong* had seen what he believed to be hard cancer in persons who presented no evidences of age or of decay.

*Prof. Hamilton* replied, that one organ might be old, in a certain sense, while other organs were still young. That the hair was often old and fell off, or became white, while the stomach and heart and other vital organs presented all of their original vigor. In further evidence of the probable correctness of his opinions, he adverted to the fact that even in epithelial cancer, occurring usually upon the skin, there was first, and generally after the age of forty, only here and there a tawny spot upon the skin of the face, then a yellowish crust, and finally an ulcer, the whole progress of the cancer from the beginning appearing very much as if the skin was ceasing its functions of growth and repair. Now the breast of the female becomes diseased with cancer usually at the very period when its functions cease, and there being no longer any occasion for its repair it would be most likely to fall into this condition of degeneration; so also the uterus was prone to cancer at the same period. These organs were now old, relatively speaking, and according to his theory ought to be, as they actually are, of all parts of the female, most prone to cancer.

*Prof. Rochester* remarked that unfortunately for Dr. Hamilton's theory, very old people were not so prone to cancer as persons of the age of forty or forty-five, or a little past the prime of life.

*Prof. Hamilton* thought this the most serious objection to his doctrine which had been mentioned; yet it was, perhaps, a question whether in proportion to the number of females living at these periods, cancer was not as frequent in extreme old age as at an earlier period; very few lived to extreme old age, and therefore, perhaps, very few had cancer at this period.

*Dr. Flint, Jr.*, had supposed that there always existed a natural predisposition to cancerous disease, hereditary or otherwise, which in cancer, did not develop itself until after the age of thirty. That the ground taken by Prof. Hamilton was undoubtedly correct in regard to schirrus being confined to old age, but that there was superadded, cachexia which determined the wearing out into this channel. That it was undoubtedly a disease of nutrition, always occurring after the age of 30.

*Prof. Hamilton* replied that he would not deny hereditary predisposition. He knew it was sometimes hereditary. But it was the same with the arcus senilis. It, too, was hereditary, for he had seen the arcus senilis occur in successive members of the same family at unusually early periods; yet it was certainly generally a disease of age, an evidence of that degeneration which belongs to age. In no other sense did he regard cancer as hereditary. Nor must it follow that all persons would have cancer who grew to be old, since it required, undoubtedly, a peculiar tendency to this degeneration, just as to have the fatty dedegeneration in some families more than in others, required a peculiar tendency.

With this compromise Dr. Strong wonld accept of Dr. Hamilton's theory.

*Prof. Flint* mentioned a case illustrating an occasional effect which feather beds had in producing asthma. The patient happened to be so situated as not to be able to sleep in her usual bed. She was immediately seized with great difficulty in breathing, which progressed to such an extent as to become very alarming. This continued for over a week, the symptoms being aggravated daily. Dr. Flint, who had been out of town, returned and had reason to suspect that the difficulty was due to the feather bed, as he found her sleeping upon one, and ascertained that it had not been her custom. On being moved to a matrass, the symptoms abated, and she soon recovered. He had first noticed this idiosyncracy in his own person. He had found that whenever he happened to be sleeping in the country, he suffered from difficulty of respiration, and was led at last to attribute it to feather beds. A striking example occurred at Louisville where he was affected in the same way for a number of nights, and at last discovered a feather bed beneath the matrass, which had escaped his search.

*Dr. Lemon* then read a report of the following case:

### *Recovery of a Child apparently Still-born, after Dr. Marshall Hall's Method. Breech Presentation.*

I beg leave to present to the society the report of a case, illustrating, as I suppose, the efficiency of Marshall Hall's method of treating "suspended respiration" from drowning, and children apparently still-born.

May 13th, at 1, P. M., I saw the wife of Timothy Duggan, Exchange st., aged about 26 years, of very delicate habit, in labor with her second child; she had been delivered of her first child with the forceps. Labor pains set in at 11, A. M.; the midwife said that the membranes ruptured at 12½. On examination I found a considerable discharge of meconium, and the "os uteri entirely dilated. It turned out to be a "breech presentation," with the "left sacro-iliac anterior position."

The expulsive pains were strong, and the trunk of a female child was born at 2, P. M.; the arms were in the usual position on the breast, the chin caught by the anterior commissure of the perineum; the pains ceased and the child remained in this position a considerable length of time, probably over fifteen minutes, the cord being pressed upon, the pulsation gradually became weaker, until I could not feel them, having got my finger into the infant's mouth, gentle traction, assisted flexion of the head as soon as the pains were renewed, and it was quickly expelled. The child was apparently dead, its surface very much congested, and of a livid color. Having tied and cut the cord, I laid the body on its face on a table, opposite an open window, and proceeded according to the directions of Marshall Hall. Ten minutes elapsed, when it began to gasp; in ten more it cried, though quite feebly.

The transition from the dark livid, to the rosy hue of the newly born infant, was quite striking and beautiful. Both mother and child did well ultimately.

*Dr. Wyckoff* mentioned a case of hæmorrhage from the umbilicus, occurring ten days after birth. It commenced the day before, and was not arrested till evening. He inquired of the members what means they had found most effectual in arresting hæmorrhage of that kind.

*Prof. Rochester* had heard a report read by Dr. Jenkins, before the Pathological Society of New York. Tne large majority of his cases terminated fatally; he used various astringents, needles and ligatures, and caustics.

*Dr. Flint, Jr.*, asked if it would always be possible to distinguish between a fracture and a cut of the umbilical cord; and also if a woman who had borne children, would always be able to distinguish between labor pains and colicy pains.

*Drs. Hamilton, Treat* and *Wilcox* thought that women were not always able to make such a distinction, and mentioned illustrative cases.

*Prof. Rochester* mentioned an action taken by the Suffolk Co. Medical Society, recommending to practitioners to present their bills quarterly. He thought the example should be followed by the Buffalo Medical Association. He therefore moved that the chair appoint a committee of three to take into consideration the advisability of such a movement and to consult with the regular practitioners of the city.

This motion was seconded, and after some discussion carried unanimously.

The chair appointed Drs. Rochester, Flint and Eastman, as such committee.

On motion, the Association then adjourned.

AUSTIN FLINT, Jr., M. D.,
Secretary.

